# Integrating Population Variants and Protein Structural Analysis to Improve Clinical Genetic Diagnosis and Treatment in Nephrogenic Diabetes Insipidus

**DOI:** 10.3389/fped.2021.566524

**Published:** 2021-04-29

**Authors:** Panli Liao, Tianchao Xiang, Hongxia Li, Ye Fang, Xiaoyan Fang, Zhiqing Zhang, Qi Cao, Yihui Zhai, Jing Chen, Linan Xu, Jialu Liu, Xiaoshan Tang, Xiaorong Liu, Xiaowen Wang, Jiangwei Luan, Qian Shen, Lizhi Chen, Xiaoyun Jiang, Duan Ma, Hong Xu, Jia Rao

**Affiliations:** ^1^Department of Nephrology, National Pediatric Medical Center of China, Children's Hospital of Fudan University, Shanghai, China; ^2^Tongji Medical College, Wuhan Children's Hospital, Wuhan Maternal and Child Healthcare Hospital, Huazhong University of Science and Technology, Wuhan, China; ^3^Shanghai Kidney Development and Pediatric Kidney Disease Research Center, Fudan University, Shanghai, China; ^4^Shanghai Key Lab of Birth Defect, Children's Hospital of Fudan University, Shanghai, China; ^5^Department of Nephrology, Beijing Children's Hospital Affiliated to Capital University of Medical Science, Beijing, China; ^6^Department of Pediatric, The First Affiliated Hospital of Sun Yat-sen University, Guangzhou, China; ^7^Key Laboratory of Metabolism and Molecular Medicine, Ministry of Education, Department of Biochemistry and Molecular Biology, School of Basic Medical Sciences, Institutes of Biomedical Sciences, Fudan University, Shanghai, China; ^8^State Key Laboratory of Medical Neurobiology, School of Basic Medical Science, Institute of Brain Science, Fudan University, Shanghai, China

**Keywords:** nephrogenic diabetes insipidus, AVPR2, AQP2, protein structure, genomics

## Abstract

Congenital nephrogenic diabetes insipidus (NDI) is a rare genetic disorder characterized by renal inability to concentrate urine. We utilized a multicenter strategy to investigate the genotype and phenotype in a cohort of Chinese children clinically diagnosed with NDI from 2014 to 2019. Ten boys from nine families were identified with mutations in *AVPR2* or *AQP2* along with dehydration, polyuria–polydipsia, and severe hypernatremia. Genetic screening confirmed the diagnosis of seven additional relatives with partial or subclinical NDI. Protein structural analysis revealed a notable clustering of diagnostic mutations in the transmembrane region of *AVPR2* and an enrichment of diagnostic mutations in the C-terminal region of *AQP2*. The pathogenic variants are significantly more likely to be located inside the domain compared with population variants. Through the structural analysis and *in silico* prediction, the eight mutations identified in this study were presumed to be disease-causing. The most common treatments were thiazide diuretics and non-steroidal anti-inflammatory drugs (NSAIDs). Emergency treatment for hypernatremia dehydration in neonates should not use isotonic saline as a rehydration fluid. Genetic analysis presumably confirmed the diagnosis of NDI in each patient in our study. We outlined methods for the early identification of NDI through phenotype and genotype, and outlined optimized treatment strategies.

## Introduction

The congenital form of nephrogenic diabetes insipidus (NDI), a rare inherited disorder, is characterized by insensitivity of the distal nephron to the antidiuretic action of arginine-vasopressin (AVP) and the reduced ability of the kidney to concentrate the urine, leading to severe dehydration and electrolyte imbalance (hypernatremia and hyperchloremia) (1). In 90% of patients, inheritance of NDI arises from mutations in the X-linked gene coding for the vasopressin type 2 receptor (*AVPR2*) (OMIM #304800) (1, 2). The remaining patients display autosomal recessive or dominant forms of inheritance due to mutations in the gene coding for the aquaporin 2 (*AQP2*) water channel (OMIM #222000; OMIM #125800, respectively) (1, 3). The main clinical hallmarks of NDI are polyuria and compensatory polydipsia. When encountering an inadequate water supply, a hot environment, or episodic losses of free water, patients suffering from NDI do not properly compensate for water loss and are at risk of severe dehydration. The defect in the ability to concentrate urine is present at birth, and is accompanied by additional symptoms that arise during the first week of life such as irritability, poor feeding, and failure to thrive (4). Persistent polyuria can lead to the development of kidney megacystis, hydroureter, and hydronephrosis. Repeated episodes of dehydration can cause cognitive difficulties (5), one of the most serious complication of NDI, probably secondary to hypoxic episodes (6). Establishing the genetic diagnosis is particularly important for NDI in order to enable early detection and a more efficient differential diagnosis in view of its unique associated features and long-term complications.

Few studies described the spectrum and prognosis of NDI from pediatric patients in China. In the current study, we utilized a multicenter strategy to investigate the genotype and phenotype in a cohort of Chinese children with NDI to explore the diagnosis and treatment for NDI in China. The description of the clinical and genetic spectrum of NDI will substantially help to devise a population-specific strategy for gene analysis.

## Materials and Methods

### Study Design and Participants

The national multicenter registry (Chinese Children Genetic Kidney Disease Database, CCGKDD) genetically screened a cohort of pediatric renal disease patients in China to date (7). Participants in this study were solicited via clinicians collaborating with CCGKDD. Participants were asked to provide information concerning their presenting clinical features, genetic diagnosis, monitoring, medical management, age at each clinical event, and clinical status at their latest follow-up. The responding clinicians were contacted to report details of clinical features, treatment, and follow-up of patients with NDI. No identifying information was collected about patients or respondents, and the names of the reporting centers were also collected to allow the comparison of entries in order to avoid duplicates. Among the children with renal disease consecutively enrolled in the national multicenter registry CCGKDD from 2014 to 2019, patients were diagnosed with NDI based on symptoms, biochemistry disturbances genetic testing, as well as water restriction/desmopressin (DDAVP) loading tests.

### Genetic Analysis

In order to further identify and confirm the diagnosis of NDI, we performed the trio whole exome sequencing (Trio-WES) with the probands and their parents concurrently. Informed consent was obtained from the parents prior to genetic analysis. WES was outsourced, and raw data were then transferred to our lab for bioinformatics analysis. FastQC software was used to examine FastQ raw data quality in terms of length of reads, GC content of reads, quality of nucleotides within the reads, and over-represented sequences, among others. Next, sequences were aligned to the Hg19 reference genome and then assessed for variant calling using the HaplotypeCaller tool of GATK software. Lastly, variants were annotated for their predicted effects on protein function and allele frequency using the public databases gnomAD (https://gnomad.broadinstitute.org/), and predicted pathogenicity was also predicted using *in silico* algorithms provided by the online software SIFT (https://sift.bii.a-star.edu.sg/sift-bin/), PolyPhen-2 score (http://genetics.bwh.harvard.edu/cgi-bin/pph2), and MutationTaster (http://www.mutationtaster.org/cgi-bin/). Diagnostic variants were defined as pathogenic or likely pathogenic according to the ACMG guidelines and also included variants of uncertain significance (VUS) of known disease-causing genes through discussion combined with genotype and phenotype. Evidence for disease causality was assessed using ClinVar (https://www.ncbi.nlm.nih.gov/clinvar/) and the Human Genome Mutation Database (HGMD; http://www.hgmd.cf.ac.uk/ac/all.php), and a manual review of primary literature. After *AVPR2* or *AQP2* mutations were identified, appropriate genetic testing and counseling were offered to symptomatic individuals, high-risk siblings, and offsprings.

### Protein Structural Analysis

The structural analysis of AVPR2 and AQP2 was performed using PDB accession 6U1N and 4NEF, respectively. The utility of Protter (https://wlab.ethz.ch/protter/start/) online software was used for the integrated visual analysis of membrane proteins (8), and residue depth was calculated computationally using DEPTH server (http://cospi.iiserpune.ac.in/depth/) (9). Population variation in *AVPR2* or *AQP2* was investigated using gnomAD and was presumed to be negligible with respect to the relative enriched and depleted pathogenic variants for rare childhood disease. According to the estimated prevalence of pediatric diabetes insipidus in males, variation in allele frequency of over 8.8/1,000,000 was described as population single-nucleotide polymorphisms (SNPs) (2). The number of residues involved in variants within the functional domains was compared with population SNPs and disease-causing mutations reported in the HGMD and by ClinVar analyses (Supplementary Table 2).

## Results

### Patient Characteristics

A total of 10 boys from nine families with registry information on CCGKDD database from 2014 to 2019 were enrolled in this study. Patient characteristics are shown in Table 1. The median age at clinical diagnosis was 1.0 months (IQR, 0.16–18). Five patients were clinically diagnosed during the neonatal period whereas three of the cases included children with delayed diagnoses who were only clinically diagnosed after 2 years old. One of the three children was identified with the mutation of *AQP2* until developing into stage 3 chronic kidney disease (CKD) at 14 years old. Polyuria–polydipsia and hypernatremia were present in all 10 probands. Fever, vomiting, and anorexia were present in the initial presentation of four of the patients. Five families had multiple effects, and only one family applied for early genetic screening for newborn babies because of his affected siblings with NDI. Five of the patients underwent water restriction tests prior to genetic analysis and eight patients underwent cranial MRI as part of their diagnostic evaluation with no reported abnormalities. Hydronephrosis was found in four children. None of the patients had hypercalciuria or abnormal renal function in serum chemistry analysis from initial presentation.

**Table 1:** Mutations and clinical follow-up of patients with nephrogenic diabetes insipidus.

**ID**	**Gender**	**Age of clinical diagnosis/Age of genetic diagnosis**	**Family history**	**Pathogenic gene**	**Mutation (Exon/Nucleotide change/Amino acid change)**	**Initial presentation**	**Urological involvement**	**Urine specific gravity/Urine volume (ml/kg.h)**	**Serum sodium/Urine sodium (mmol/L)**	**Pituitary gland MRI**	**Water deprivation test**	**Treatment/Last follow-up (serum Na: mmol/L, age: years)**
1	Male	3.6 years/4 years	None	***AQP2***	exon3:c.559C>T; p.R187C (HOM)	Polydipsia, polyuria	None	<1.005/20	159/20	Normal	Negative	Thiazide/145, 6.5
2	Male	3 years/13 years	Paternal/Paternal grandpa	***AQP2***	exon4 c.803delG p.R268Vfs^*^67 (het, p, het; m, wt)	Polydipsia, polyuria	None	<1.005/23	150/N.D.	N.D.	N.D.	Thiazide/140, 14.2^#^
3	Male	5 days/2 months	Maternal uncle	***AVPR2***	exon2:c.185T>C; p.L62P (Hemi)	Jaundice fever, vomiting, anorexia, hypernatremia	None	<1.005/12	161/54	Normal	Negative	Thiazide, indometacin/145, 4.2
4	Male	3 days/26 days	None	***AVPR2***	exon1:c.229-231delTTC; p.F77del (Hemi)	Polyuria, fever, vomiting, anorexia	Hydronephrosis	<1.005/10	162/16	Normal	N.D.	Thiazide, indometacin/145, 2.5
5	Male	2 months/2 months	None	***AVPR2***	exon2:c.494C>A; p.A165D (Hemi)	Fever, vomiting, anorexia	None	<1.005/7	162/14	Normal	N.D.	Thiazide/148, 3.0
6	Male	2 years/4.1 years	None	***AVPR2***	exon3:c.500C>T; p.S167L (hemi)	Polydipsia, polyuria	None	1.002/7	145/26	Normal	Negative	Thiazide, indometacin/140, 7.5
7–1^*^	Male	1 year/1 year	Maternal	***AVPR2***	exon3:c.500C>T; p.S167L (Hemi)	Polydipsia, polyuria	None	1.000/7.4	145/23	Normal	Negative	Thiazide/140, 2.2
7–2^*^	Male	14 days/1 months	Maternal/maternal grandpa	***AVPR2***	exon3:c.991-992delGC; p.S331Rfs^*^25 (Hemi)	Polydipsia, polyuria, fever, vomiting, anorexia	Hydronephrosis	1.000/9	149/12.2	Normal	N.D.	Thiazide/142, 10.5
8	Male	1 month/2 months	Maternal/maternal grandpa	***AVPR2***	exon3:c.991-992delGC; p.S331Rfs^*^25 (Hemi)	Polydipsia, polyuria, fever, vomiting, anorexia	Hydronephrosis	1.003/10	160/48	Normal	N.D.	Thiazide, indometacin/145, 2.6
9	Male	1 month/4.6 years	Maternal uncle	***AVPR2***	Exon2 del (Hemi)	Polydipsia, polyuria, urinary incontinence	Hydronephrosis	1/15	135/17	Normal	Negative	Thiazide/135, 5.8

*All patients with mutations in the AVPR2 gene were hemizygous segregated with all mothers were heterozygous for the given mutation*.

*het, heterozygous; hemi, hemizygous; m, maternal; N.D., no data; p, paternal*;

* *siblings*.

# *the patient developed into CKD 3 stage at 14 years old*.

### Genetic Analysis

**Figure 1:**
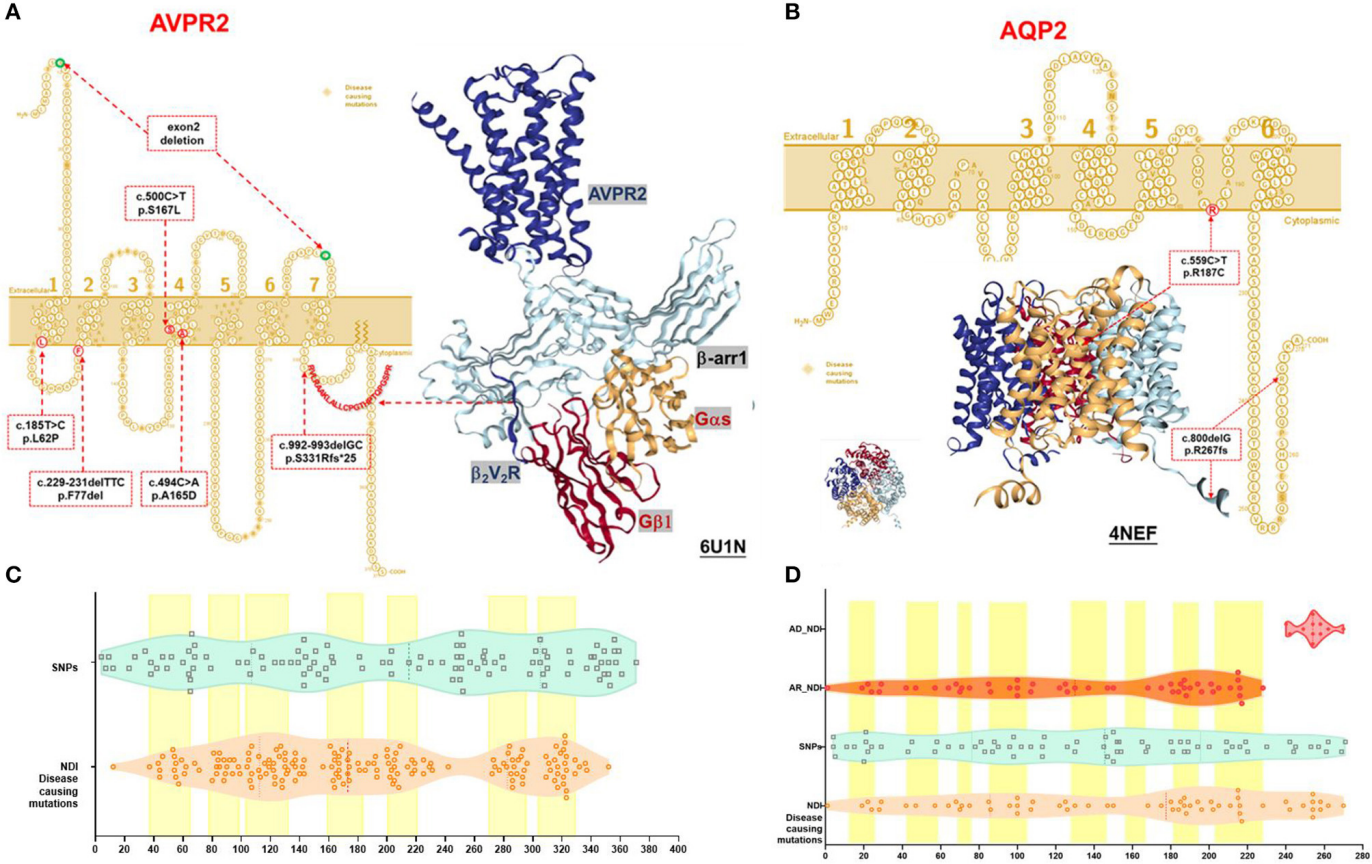
Schematic representation and protein structure of AVPR2 and AQP2 protein with location of mutations. **(A)** AVPR2, which belongs to the G-protein-coupled receptor (GPCR) superfamily, is formed by 371 amino acids with seven transmembrane, four extracellular, and four cytoplasmic domains, and is present at the basolateral membrane (UniProtKB P30518-1, P30518-2) (14). The seven transmembrane domains with the extracellular NH2 terminus and its intracellular COOH terminus are illustrated as originally reported. Ribbon diagram of AVPR2 from PDB entry 6U1N was shown with GPCR-β arrestin structure in lipid bilayer. The six mutations identified in our NDI cohort were indicated in the red dotted box, and the diagnostic mutations by HGMD were labeled in the orange diamond. **(B)** AQP2, which belongs to the larger family of major intrinsic proteins, is formed by 271 amino acids. Aquaporins contain two tandem repeats, each containing three transmembrane α-helices domains with the amino and the carboxyl termini located on the cytoplasmic surface of the membrane (UniProtKB P41181) (15). The six transmembrane domains with the intracellular NH2 and COOH terminus are illustrated as originally reported. Ribbon diagram of AQP2 from PDB entry 4NEF was shown with side and top view. The two mutations identified in our NDI cohort were indicated in the red dotted box, and the diagnostic mutations by HGMD were labeled in the orange diamond. **(C)** A violin plot revealed the domain composition of AVPR2 with the location of the 109 population variants (SNPs) from gnomAD (turquoise square) and the 169 diagnostic mutations from HGMD (peachpuff circle). The seven transmembrane regions were labeled as a yellow box. There was a significant enrichment of diagnostic mutations in the transmembrane regions (Fisher test, *P* = 0.001). **(D)** A violin plot revealed the domain composition of AQP2 with the location of the 70 population variants (SNPs) from gnomAD (turquoise square) and the 62 diagnostic mutations from HGMD (peachpuff circle) including 56 mutations inherited by autosomal recessive trait (AR, orange circle) and 6 mutations inherited by autosomal dominant trait (AD, scarlet dot). The eight transmembrane regions were labeled as a yellow box. There was no significant enrichment of diagnostic mutations in transmembrane region compared with population variants (Fisher test, *p* = 0.079). The mutations by AD were more likely located in the C-terminal region compared with that by AR (Pearson test, *p* = 0.0001).

We detected six different mutations of *AVPR2* and two mutations of *AQP2* in the study cohort. Four of these mutations were previously reported mutations [*AVPR2*: p.L62P (10), p.A165D (11), and p.S167L (12); *AQP2*: p.R187C (13)], and four novel mutations were observed (*AVPR2*:p.F77del, p.S331Rfs^*^25, and small deletion of exon2; *AQP2*: p.R267fs^*^66). In total, we found four missense mutations, three small deletions resulting in frameshift mutations and a gross deletion of an exon (Table 1 and Figure 1). Pathogenicity predictions by *in silico* algorithms are shown in Supplementary Table 1.

We also detected seven female relatives with heterozygous mutations and two maternal uncles with hemizygous mutations in the *AVPR2* gene. All relatives reported multiple episodes of mild or moderate fever during childhood without any differential diagnosis details on dehydration fever or infection disease. Among them, we found that three suffered polydipsia and polyuria from childhood, and four female individuals presented partial or subclinical NDI. However, further examination has not been done. They all had multiple episodes of mild or moderate fever during childhood. Additionally, one maternal uncle was aware that he had a lack of thirst despite a large fluid intake.

### Variant Analysis and Comparative Protein Modeling

**Figure 2:**
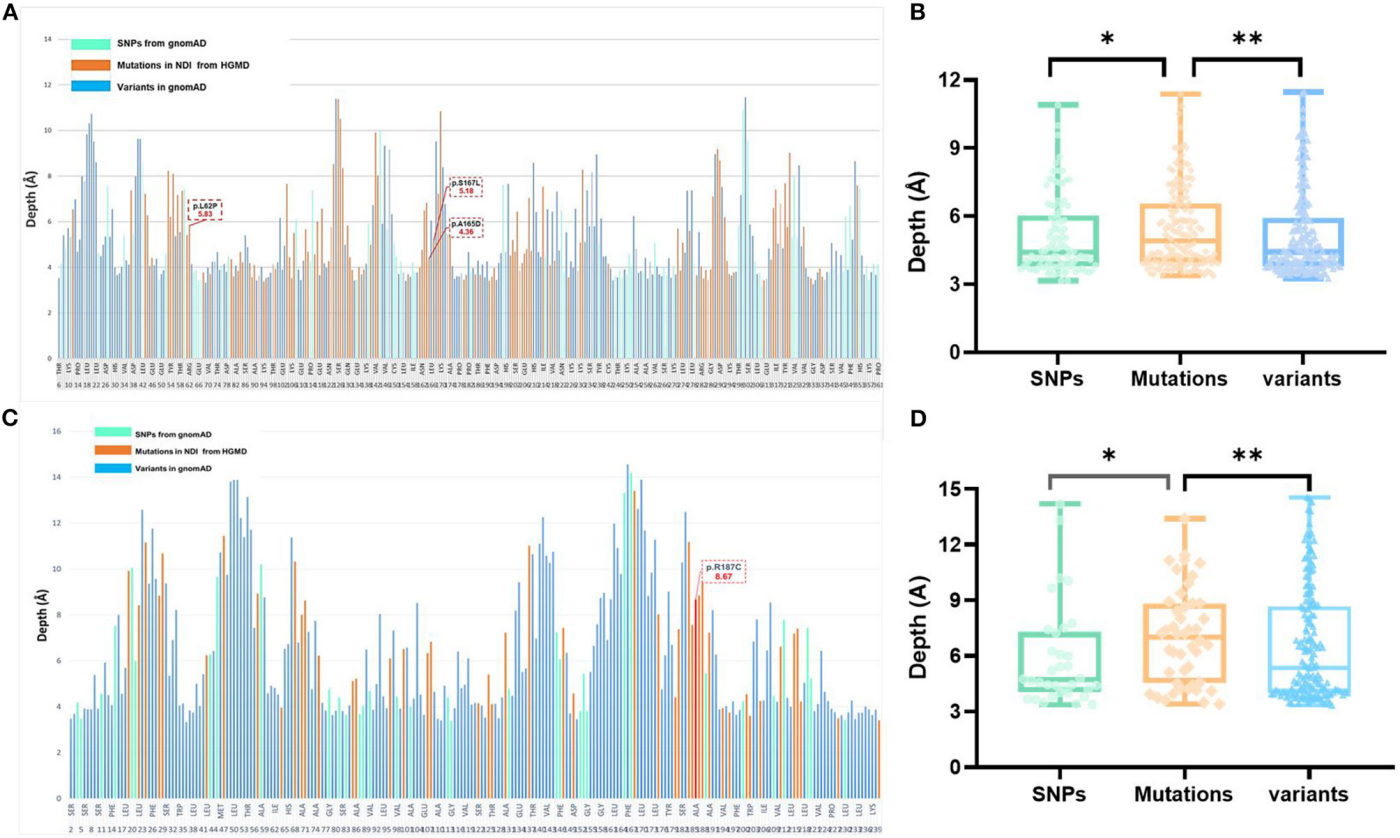
Analysis of variants and residue depth computation in AVPR2 and AQP2. **(A)** A 2D plot of residue-wise depth of AVPR2 (PDB 6U1N) with bars indicating the diagnostic mutations by HGMD (orange), population variants/SNPs by gnomAD (turquoise), and all the other residues (blue). **(B)** Boxplot of the residue depth (9) comparing the diagnostic mutations (orange), population variants/SNPs (turquoise), and all the other residues (blue) in AVPR2. *p = 2e^−8^, **p = 2e^−5^ (Wilcoxon–Mann–Whitney test). **(C)** A 2D plot of residue-wise depth of AQP2 (PDB 4NEF) with bars indicating the diagnostic mutations by HGMD (orange), population variants/SNPs by gnomAD (turquoise), and all the other residues (blue). **(D)** Boxplot of the residue depth comparing the diagnostic mutations (orange), population variants/SNPs (turquoise), and all the other residues (blue) in AQP2. *p = 2e^−7^, **p = 2e^−5^ (Wilcoxon–Mann–Whitney test).

The AVPR2 protein is a typical seven-membrane-spanning helix G protein-coupled receptor (GPCR) localized at the basolateral plasma membrane of the principal cells of the kidney collecting duct (14). In available literature, we found 169 reported missense mutations in *AVPR2* in patients with NDI, and there are a further 109 population missense variants retrieved from the gnomAD database. The location of disease-causing and population variants was scattered throughout the different structural domains of the protein, with significant enrichment of disease-causing variants in the transmembrane region (Fisher test, *p* = 0.001, Figures 1A,C). The residue depth of the disease-causing mutation is significantly higher than that of the population SNPs and that of all variants from gnomAD (Wilcoxon–Mann–Whitney test, *p* = 2e^−8^ and *p* = 2e^−5^, respectively, Figures 2A,B).

Structural analysis of the AVPR2 protein revealed the presence of hemizygous mutations in eight patients (Figure 1A). The three missense mutations of *AVPR2* (p.L62P, p.A165D, and p.S167L) produce full-length misfolded proteins with residues located within the transmembrane domain. The protein is mostly retained in the endoplasmic reticulum (ER) by the ER quality control machinery and is target for proteasome degradation. The deletion mutation p.F77del resulted in the residue deficiency in the first intracellular loop (located in a well-conserved region), likely causing a defective protein. The small deletion p.S331Rfs^*^25 caused a frameshift with a premature stop codon encountered in the sequence. This deficiency of the C-terminal tail of AVPR2 results in the loss of β-arrestin binding to the phosphorylated tail of AVPR2 and subsequent receptor internalization, which would normally allow for the engagement of G protein to the core of AVPR2. The formation of megaplex AVPR2–G protein–β-arrestin is required for the active signals leading to sustained endosomal cAMP generation (16). The gross deletion of exon 2 led to truncated proteins, which are often rapidly degraded.

The *AQP2* gene is located in the *12q13* region and codes for the 271-amino-acid AQP2 protein, a type IV-A transmembrane protein characterized by six transmembrane domains connected by five loops and intracellular N- and C-termini (15). A total of 53 missense mutations and 9 small deletions in *AQP2* were reported, and there were a further 70 population missense variants retrieved from the gnomAD database. The location of disease-causing and population variants was scattered throughout the different structural domains of the protein, with no significant enrichment of pathogenic variants in the transmembrane regions (Fisher test, *p* = 0.079, Figure 1B). However, five of the nine small deletions in AQP2 affected the residues from 223 to 271 in the C-terminal cytoplasmic region. There was a significant enrichment of pathogenic variants by autosomal dominant inheritance (AD) in the C-terminal region (Pearson test, *p* = 0.0001, Figure 1D). The residue depth of the pathogenic variants is significantly higher than that of the population SNPs and that of all variants from gnomAD (Wilcoxon–Mann–Whitney test, *p* = 2e^−7^ and *p* = 2e^−5^, respectively, Figures 2C,D).

Structural analysis of the AQP2 protein revealed the presence of mutations in two patients (Figure 1B). The homozygous mutation p.R187C affected amino acids in the selectivity filter region of the water conduction pore, which determines the transport specificity (17). ER accumulation of these AQP2 mutants has been shown in another study (4). The C-terminal truncating mutation p.R267fs^*^66 could result in the heterotetramers formed by wild type and mutated AQP2 monomers that were either retained in the Golgi apparatus or misrouted to late endosomes, lysosomes, or basolateral membrane. This type of mutation is inherited from a dominant trait in NDI (18).

### Treatment Regimens

**Figure 3:**
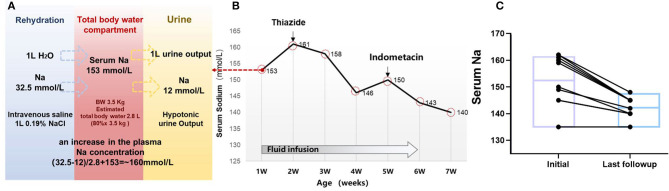
Clinical treatment and outcome in 10 children with NDI. **(A)** Simplified tonicity balance for the neonate ID#3 with NDI, excreting hypotonic urine and receiving 0.19% saline. When considering separately the balances for water and Na, the excess Na administration from 0.19% saline becomes immediately obvious. The red box in the middle represents the total body water compartment of a patient with NDI. A patient with NDI excretes hypotonic urine, detected with the urine Na concentration of 12 mmol/L. If 1 L of urine output is replaced with 1 L of 0.19% saline, this will not change the fluid balance but lead to a net gain of 20.5 mmol of Na. In the neonate of 3.5 kg with estimated 2.8 L of total body water, this would lead to an increase in the plasma Na concentration of ~20.5 mmol/2.8 L = 7.3 mmol/L. **(B)** Serum sodium and treatment in the case #3. **(C)** Follow-up of the serum sodium level in 10 patients with treatment of thiazides and indometacin.

All the patients diagnosed with NDI as neonates required emergency treatment of hypernatremia dehydration in this study. Most patients received isotonic or hypotonic rehydration before being clinically diagnosed with NDI. For example, this was observed in our case example ID#3, where plasma sodium level increased from 153 to 161 mmol/L after rehydration with intravenous 0.19% saline for the neonate. A tonicity balance can easily demonstrate the excess of NaCl administration from 0.19% saline (Figures 3A,B). In this case, the appropriate concentration of 5% glucose was given, which shows that the free water loss was prescribed for maintenance fluid rates appropriate to his age and size, and adjustment according to the plasma sodium. Breastfeeding was preferable to formula feed for its hypotonic advantage.

Most of the patients in this study started the conventional treatment with thiazides after clinical diagnosis of NDI, followed by non-steroidal anti-inflammatory drugs (NSAIDs). Thiazides were prescribed in five patients, whereas thiazides and NSAIDs were prescribed in four of the patients. During the follow-up period, NSAIDs were discontinued for the following reasons including general concerns about long-term use in the three of the patients, and increased serum creatinine levels in one of the patients. The medium serum sodium at initial treatment was 154 mmol/L (IQR, 145–161) and was reduced to 144 mmol/L (IQR, 140–145) at the last follow-up, with a median age of 5 years (IQR, 2.6–8.3) (Figure 3C). At the time of last follow-up, only one case (#_2) with *AQP2* mutation developed into CKD 3 stage. It is noteworthy to mention that the father and maternal grandfather of case #2 passed away with renal failure.

## Discussion

This study reported the genetic spectrum and treatment approaches in a cohort of children with NDI registered on the Chinese multicenter database. We reported eight cases from seven families presented with X-linked NDI (*AVPR2*); one case presented with autosomal recessive NDI (*AQP2*) and one case presented with autosomal dominant NDI (*AQP2*).

Although the pathophysiology and molecular diagnosis of congenital polyuric states have been well-established (19), we still encountered cases where the diagnoses were late and where inappropriate diagnostic testing and treatments are performed. Optimizing the diagnostic strategy especially with regard to genetic analysis was one of our top priorities. It has been well-established that the heterozygous loss-of-function variants o*f AVPR2* or *AQP2* are common disease-causing genes that result in congenital NDI (2, 3, 20). In order to try to elucidate the pathogenicity of variants of *AVPR2* or *AQP2*, we compared population SNPs with pathogenic mutations from the HGMD. Although the existence of a single population variant does not rule out pathogenicity, it is unlikely that the observed population variants of *AVPR2* or *AQP2* are pathogenic, since severe early onset childhood disorders have specifically been excluded from gnomAD. Therefore, we evaluated the variants in the 3D domain structure encoded by AVPR2 or AQP2 to determine positional correlation with pathogenicity. There was notable clustering in the AVPR2 3D structure, with pathogenic mutations more likely to be within the transmembrane region. Such clustering in the transmembrane region was not shown for AQP2. An enrichment of diagnostic mutations by autosomal dominant inherited (AD) was found in the C-terminal region of *AQP2*. Nonetheless, the pathogenic missense mutations of *AVPR2* or *AQP2* were significantly more likely to be located within the domain. Systemic analysis of the protein structure and variants allowed us to make strong predictions about likely pathogenic variations in both *AVPR2* and in *AQP2. One* of the limitations of our study is the lack of functional studies, especially in case of the novel variants detected in the NDI cohort. Thus, the mutations identified in our study were considered as presumably disease-causative post from the protein structural analysis and *in silico* predictions.

As next-generation sequencing is increasingly applied in both research and clinical settings, more and more variants will be discovered in known disease-causative genes as well as in novel genes. Although *in silico* predictions alone should not be relied on as the sole basis to determine the clinical significance of variants in proteins, we hope that the findings of this study provide useful structural evidence for variant interpretation. Moreover, combining clinical and population genetics with protein structural analysis offers widely applicable *in silico* methods for improving the clinical interpretation of novel missense variations.

In patients with NDI, high fluid intake is necessary to avoid hypernatremia dehydration, which can otherwise result in permanent neurologic complications. Most emergency protocols suggest an initial treatment with 0.9% saline (21). However, the situation with NDI is different because of the ongoing loss of pure water into urine. We described the clinical case with an infusion of 0.19% saline resulting in excess sodium chloride administration and thus worsening the hypernatremia. Thus, children with NDI should be treated with hypotonic fluids, either enterally with water or milk as shown in case #3, or, if need be, intravenously with 5% dextrose in water (6, 22). The diagnosis of congenital NDI can neither be missed nor misunderstood, as it would lead to dangerous mistreatment. The early identification of NDI through phenotypes and genotypes is important, as is treatment optimization.

Hypernatremia in children with NDI will induce a strong thirst behavior. When asked, the parents of patients with NDI often described the typical attributes of an extremely thirsty child, one who so avidly drinks large amounts of water, and often vomits afterward. In our cohort, five neonates were diagnosed early with symptoms including fever, vomiting, and anorexia. Five families had multiple members previously diagnosed with NDI; however, only one family had applied for early genetic screening for their newborn due to having siblings already diagnosed with NDI. In addition to the probands, we identified a further three males diagnosed with NDI and four female individuals who presented partial or subclinical NDI by sequencing *AVPR2* and *AQP2*. Further examination found that those affected had frequent episodes of mild or moderate fever during childhood and a lack of thirst in adulthood. Confirming the clinical diagnosis of NDI with genetic screening allows for the early diagnosis and management of at-risk members of families with identified mutations (6, 19, 23). NDI is a rare disease and does not prominently feature on the diagnostic radar of frontline medical staff. The medical attention for the undiagnosed children with a fever of unknown etiology or large urine output, combined with further investigation of hypernatremia with inappropriately diluted urine, is sufficient to trigger the diagnosis of NDI. For families with suspected NDI affects, appropriate genetic testing and counseling should be offered to the symptomatic individuals, high-risk siblings and offspring, and pregnant woman.

Our study had several limitations. First, skewed X-chromosome inactivation was not checked in the female carriers screened in our study. It has been shown that a frequency of about 25–50% of NDI exists in female carriers ^2, 3.^ Additionally, data were collected retrospectively from the registry system and we were not able to obtain complete data on all of the patients. We described the complications and treatment approaches of NDI during a median follow-up period of 5 years. Urological complications such as hydronephrosis and urinary incontinence were noted in 4 of 10 individuals. Unfortunately, there were no details on nutrition, growth, and mental development in our registry. It has been reported that the long-term morbidities caused by NDI include primary nocturnal enuresis (44%), persistent small stature (38%), urologic complications (37%), persistent failure to thrive (29%), and stage 2 or greater CKD (30%) (23). Here, we reported a signal patient with delayed diagnosis who later developed into stage 3 CKD.

In conclusion, newborn and young children with polyuria symptoms should be immediately referred to specialized centers with experience in treating hypernatremia dehydration and the ability to rapidly obtain genetic analyses and provide a clinical diagnosis.

## Data Availability Statement

The data that support the findings of this study are available from the corresponding author upon reasonable request. The datasets presented in this article are not readily available because a regulation on the management of human genetic resources from the State Council, CHINA. Requests to access the datasets should be directed to the database for Chinese children renal disease which is publicly available datasets in Chinese language (https://www.ccgkdd.com.cn/).

## Ethics Statement

The studies involving human participants were reviewed and approved by The Institutional Review Board (IRB) of Children's Hospital of Fudan University (No. 2018_286). Written informed consent to participate in this study was provided by the participants' legal guardian/next of kin. The parents of the children and adult patients described in this article provided consent for participation in the study and for publishing the obtained results.

## Author Contributions

JR designed and supervised the study and wrote the manuscript. PLL and HXL performed clinical examinations, collected blood samples, and wrote the clinical part of the manuscript. TCX, YF, and JR performed bio-information evaluation and protein structural analysis. JR, HX, and DM critically revised the manuscript. All the authors contributed to the clinical information and registry database.

## Conflict of Interest

The authors declare that the research was conducted in the absence of any commercial or financial relationships that could be construed as a potential conflict of interest.

## Abbreviations

3D: three dimensions
AD: autosomal dominant inherited
AR: autosomal recessive inherited
AVP: arginine-vasopressin
AVPR2: vasopressin type 2 receptor
AQP2: aquaporin 2
CCGKDD: Chinese Children Genetic Kidney Disease Database
CKD: chronic kidney disease
DDAVP: Desmopressin
GPCR: G protein-coupled receptor
HGMD: Human Genome Mutation Database
IQR: Interquartile range
NDI: nephrogenic diabetes insipidus
NSAIDs: non-steroidal anti-inflammatory drugs
OMIM: Online Mendelian Inheritance in Man
PCR: polymerase chain reaction
SNPs: single-nucleotide polymorphism
Trio-WES: trio whole exon sequence
WES: Whole Exome Sequencing.

## References

[1] Milano S Carmosino M Gerbino A Svelto M Procino. Hereditary nephrogenic diabetes insipidus: pathophysiology and possible treatment. An update. Int J Mol Sci. (2017) 18:2385. 10.3390/ijms18112385 29125546 PMC5713354

[2] Arthus MF Lonergan M Crumley MJ Naumova AK Morin D De Marco LA. Report of 33 novel AVPR2 mutations and analysis of 117 families with X-linked nephrogenic diabetes insipidus. J Am Soc Nephrol. (2000) 11:1044–54. 10.1681/j.ISSN.1046-6673.2000.11.007 10820168

[3] Joshi S Kvistgaard H Kamperis K Faerch M Hagstrom S Gregersen N. Novel and recurrent variants in AVPR2 in 19 families with X-linked congenital nephrogenic diabetes insipidus. Eur J Pediatr. (2018) 177:1399–405. 10.1007/s00431-018-3132-z 29594432

[4] Saglar Ozer E Moeller HB Karaduman T Fenton RA Mergen H. Molecular characterization of an aquaporin-2 mutation causing a severe form of nephrogenic diabetes insipidus. Cell Mol Life Sci. (2020) 77:953–62. 10.1007/s00018-019-03219-w 31302751 PMC11104860

[5] Sharma S Ashton E Iancu D Arthus MF Hayes W Van't Hoff W. Long-term outcome in inherited nephrogenic diabetes insipidus. Clin Kidney J. (2019) 12:180–7. 10.1093/ckj/sfy027 30976394 PMC6452213

[6] Bockenhauer D Bichet DG. Nephrogenic diabetes insipidus. Curr Opin Pediatr. (2017) 29:199–205. 10.1097/MOP.0000000000000473 28134709

[7] Rao J Liu X Mao J Tang X Shen Q Li G. Genetic spectrum of renal disease for 1001 Chinese children based on a multicenter registration system. Clin Genet. (2019) 96:402–10. 10.1111/cge.13606 31328266

[8] Omasits U Ahrens CH Muller S Wollscheid B. Protter: interactive protein feature visualization and integration with experimental proteomic data. Bioinformatics. (2014) 30:884–6. 10.1093/bioinformatics/btt607 24162465

[9] Chakravarty S Varadarajan R. Residue depth: a novel parameter for the analysis of protein structure and stability. Structure. (1999) 7:723–32. 10.1016/S0969-2126(99)80097-5 10425675

[10] Knoers NV van den Ouweland AM Verdijk M Monnens LA van Oost BA. Inheritance of mutations in the V2 receptor gene in thirteen families with nephrogenic diabetes insipidus. Kidney Int. (1994) 46:170–6. 10.1038/ki.1994.256 7933835

[11] Schulz A Sangkuhl K Lennert T Wigger M Price DA Nuuja A. Aminoglycoside pretreatment partially restores the function of truncated V(2) vasopressin receptors found in patients with nephrogenic diabetes insipidus. J Clin Endocrinol Metab. (2002) 87:5247–57. 10.1210/jc.2002-020286 12414899

[12] Wildin RS Antush MJ Bennett RL Schoof JM Scott CR. Heterogeneous AVPR2 gene mutations in congenital nephrogenic diabetes insipidus. Am J Hum Genet. (1994) 55:266–77. 7913579 PMC1918356

[13] van Lieburg AF Verdijk MA Knoers VV van Essen AJ Proesmans W Mallmann R. Patients with autosomal nephrogenic diabetes insipidus homozygous for mutations in the aquaporin 2 water-channel gene. Am J Hum Genet. (1994) 55:648–52. 7524315 PMC1918308

[14] Lolait SJ O'Carroll AM McBride OW Konig M Morel A Brownstein MJ. Cloning and characterization of a vasopressin V2 receptor and possible link to nephrogenic diabetes insipidus. Nature. (1992) 357:336–9. 10.1038/357336a0 1534150

[15] Bai L Fushimi K Sasaki S Marumo F. Structure of aquaporin-2 vasopressin water channel. J Biol Chem. (1996) 271:5171–6. 10.1074/jbc.271.9.5171 8617798

[16] Nguyen AH Thomsen ARB Cahill TJ III Huang R Huang LY Marcink T. Structure of an endosomal signaling GPCR-G protein-beta-arrestin megacomplex. Nat Struct Mol Biol. (2019) 26:1123–31. 10.1038/s41594-019-0330-y 31740855 PMC7108872

[17] Frick A Eriksson UK de Mattia F Oberg F Hedfalk K Neutze R. X-ray structure of human aquaporin 2 and its implications for nephrogenic diabetes insipidus and trafficking. Proc Natl Acad Sci USA. (2014) 111:6305–10. 10.1073/pnas.1321406111 24733887 PMC4035913

[18] Asai T Kuwahara M Kurihara H Sakai T Terada Y Marumo. Pathogenesis of nephrogenic diabetes insipidus by aquaporin-2 C-terminus mutations. Kidney Int. (2003) 64:2–10. 10.1046/j.1523-1755.2003.00049.x 12787389

[19] Bockenhauer D Bichet DG. Pathophysiology, diagnosis and management of nephrogenic diabetes insipidus. Nat Rev Nephrol. (2015) 11:576–88. 10.1038/nrneph.2015.89 26077742

[20] García Castaño A Pérez de Nanclares G Madariaga L Aguirre M Chocron S Madrid A. Novel mutations associated with nephrogenic diabetes insipidus. A clinical-genetic study. Eur J Pediatr. (2015) 174:1373–85. 10.1007/s00431-015-2534-4 25902753

[21] Santillanes G Rose E. Evaluation and management of dehydration in children. Emerg Med Clin North Am. (2018) 36:259–73. 10.1016/j.emc.2017.12.004 29622321

[22] D'Alessandri-Silva C Carpenter M Mahan JD. Treatment regimens by pediatric nephrologists in children with congenital nephrogenic diabetes insipidus: A MWPNC study. Clin Nephrol. (2018) 89:358–63. 10.5414/CN109127 29162216

[23] D'Alessandri-Silva C Carpenter M Ayoob R Barcia J Chishti A Constantinescu A. Diagnosis, treatment, and outcomes in children with congenital nephrogenic diabetes insipidus: a pediatric nephrology research consortium study. Front Pediatr. (2019) 7:550. 10.3389/fped.2019.00550 32039113 PMC6985429

[24] Liao P Xiang T Li H Fang Y Fang X Zhang Z. Integrating population variation and protein structural analysis to improve the clinical genetic diagnosis and treatment in children with congenital nephrogenic diabetes insipidus. Res Square [Preprint]. 10.21203/rs.3.rs-28437/v1 PMC8116627 33996673

