# Supplementary material for: Integrating Population Variants and Protein Structural Analysis to Improve Clinical Genetic Diagnosis and Treatment in Nephrogenic Diabetes Insipidus
Source: Front Pediatr. 2021 Apr 29;9:566524. doi: 10.3389/fped.2021.566524 (PMC8116627; doi:10.3389/fped.2021.566524)
Supplement: Supplementary file 1 [file Data_Sheet_1.pdf]

Supplementary Table 1. Pathogenicity of mutations predicted with in silico algorithms.

	AVPR2							*AQP2*	
Mutation	p.L62P (Hemi)	p.F77del	p.A165D	p.S167L	p.S167L	p.S331Rfs*25	Exon2 del	p.R187C	p.R268Vfs*67
PPH2 score	1	-	0.763	-	0.993	-	-	0.998	-
SIFT	DAMAGING	-	TOLERATED	-	DAMAGING	-	-	DAMAGING	-
MT	Disease Causing	-	Disease Causing	-	Disease Causing	-	-	Disease Causing	-
HGMD	Knoers (1994) Kidney Int 46, 170[10]	None	Schulz (2002) J Clin Endocrinol Metab 87, 5247[11]	None	Wildin (1994) Am J Hum Genet 55, 266[12]	None	None	Lieburg (1994) Am J Hum Genet 55, 648[13]	None
Ortholog	Dr	-	Mm	-	Gg	-	-	Xt	-
gnomAD (ESA)	1/177848 (0/13463)	None	None	None	None	None	None	15/282878 (0/19954)	none
ACMG	LP(PM1,PP1，PP3)	LP(PM1,PM2,PP1，PP3)	LP(PM1, PM2,PP1, PP3)	LP(PM1, PM2,PP1，PP3)	LP(PM1, PM2,PP1，PP3)	P(PVS1;PM2,PP3)	P(PVS1;PM2,PP3)	LP(PM1,PP1,PP3)	P(PVS1;PM2,PP3)

Supplementary Table 2. Disease-causing mutations reported in the HGMD

GENE	Mutation	Amino acid change	Reference
AQP2	Missense change	p.Met1Ile	Sahakitrungruang (2008) Endocrine 33, 210
AQP2	Missense change	p.Ala19Val	Sasaki (2013) Clin Exp Nephrol 17, 338
AQP2	Missense change	p.Leu22Val	Canfield (1997) Hum Mol Genet 6, 1865
AQP2	Missense change	p.Val24Ala	Leduc-Nadeau (2010) J Physiol 588, 2205
AQP2	Missense change	p.Leu28Pro	Marr (2002) J Am Soc Nephrol 13, 2267
AQP2	Missense change	p.Gly29Ser	Sahakitrungruang (2008) Endocrine 33, 210
AQP2	Missense change	p.Ala47Val	Marr (2002) J Am Soc Nephrol 13, 2267
AQP2	Missense change	p.Gln57Pro	Lin (2002) J Clin Endocrinol Metab 87, 2694
AQP2	Missense change	p.Gly64Arg	van Lieburg (1994) Am J Hum Genet 55, 648
AQP2	Missense change	p.Asn68Ser	Mulders (1997) J Am Soc Nephrol 8, 242
AQP2	Missense change	p.Ala70Asp	Cheong (2005) J Korean Med Sci 20, 1076
AQP2	Missense change	p.Val71Met	Bichet (1995) J Am Soc Nephrol 6, 717A
AQP2	Missense change	p.Arg85Term	Vargas-Poussou (1997) J Am Soc Nephrol 8, 1855
AQP2	Missense change	p.Ala86Val	GarcÃ-a CastaÃ±o (2015) Eur J Pediatr 174, 1373
AQP2	Missense change	p.Gly96Glu	Rugpolmuang (2014) J Pediatr Endocrinol Metab 27, 193
AQP2	Missense change	p.Gly100Arg	Carroll (2006) Genet Med 8, 443
AQP2	Missense change	p.Gly100Val	Lin (2002) J Clin Endocrinol Metab 87, 2694
AQP2	Missense change	p.Gly100Term	Hochberg (1997) J Clin Endocrinol Metab 82, 686
AQP2	Missense change	p.Ile107Asn	Zaki (2006) Nephrol Dial Transplant 21, 1082
AQP2	Missense change	p.Thr108Met	Park (2014) Biomed Rep 2, 596
AQP2	Missense change	p.Thr125Met	Goji (1998) J Clin Endocrinol Metab 83, 3205
AQP2	Missense change	p.Thr126Met	Mulders (1997) J Am Soc Nephrol 8, 242
AQP2	Missense change	p.Ala130Val	Fujimoto (2014) Yonago Acta Med 57, 85
AQP2	Missense change	p.Leu137Pro	Duzenli (2012) Endocrine 42, 664
AQP2	Missense change	p.Ala147Thr	Mulders (1997) J Am Soc Nephrol 8, 242
AQP2	Missense change	p.Asp150Glu	Iolascon (2007) Nephron Physiol 105, p33
AQP2	Missense change	p.Val168Met	Vargas-Poussou (1997) J Am Soc Nephrol 8, 1855
AQP2	Missense change	p.Gly175Arg	Goji (1998) J Clin Endocrinol Metab 83, 3205
AQP2	Missense change	p.Gly180Ser	Carroll (2006) Genet Med 8, 443
AQP2	Missense change	p.Cys181Trp	Canfield (1997) Hum Mol Genet 6, 1865
AQP2	Missense change	p.Asn184His	Fujimoto (2014) Yonago Acta Med 57, 85
AQP2	Missense change	p.Pro185Ala	Bichet (1995) J Am Soc Nephrol 6, 717A
AQP2	Missense change	p.Arg187His	Cheong (2005) J Korean Med Sci 20, 1076
AQP2	Missense change	p.Arg187Cys	van Lieburg (1994) Am J Hum Genet 55, 648
AQP2	Missense change	p.Ala190Thr	Bichet (1995) J Am Soc Nephrol 6, 717A
AQP2	Missense change	p.Val194Ile	Marr (2002) J Am Soc Nephrol 13, 2267
AQP2	Missense change	p.Gly196Asp	Loonen (2008) Semin Nephrol 28, 252
AQP2	Missense change	p.His201Tyr	Liberatore Junior (2012) Clinics (Sao Paulo) 67, 79
AQP2	Missense change	p.Trp202Cys	Oksche (1996) Hum Genet 98, 587
AQP2	Missense change	p.Gly211Arg	Liberatore Junior (2012) Clinics (Sao Paulo) 67, 79
AQP2	Missense change	p.Gly215Ser	Cen (2015) Int J Clin Exp Med 8, 3629
AQP2	Missense change	p.Gly215Cys	Iolascon (2007) Nephron Physiol 105, p33

AQP2	Missense change	p.Ser216Pro	van Lieburg (1994) Am J Hum Genet 55, 648
AQP2	Missense change	p.Ser216Phe	Moon (2009) Endocr J 56, 905
AQP2	Missense change	p.Lys228Glu	Leduc-Nadeau (2010) J Physiol 588, 2205
AQP2	Missense change	p.Arg254Glu	Savelkoul (2009) Hum Mutat 30, E891
AQP2	Missense change	p.Arg254Leu	de Mattia (2005) J Am Soc Nephrol 16, 2872
AQP2	Missense change	p.Arg254Trp	Dollerup (2015) BMC Nephrol 16, 217
AQP2	Missense change	p.Glu258Lys	Mulders (1998) J Clin Invest 102, 57
AQP2	Missense change	p.Pro262Leu	Bichet (1995) J Am Soc Nephrol 6, 717A
AQP2	Del	p.His67fs*39	Tajima (2003) Endocr J 50, 473
AQP2	Del	p.Leu121fs*30	van Lieburg (1994) Am J Hum Genet 55, 648
AQP2	Del	p.Leu218fs*20	Marr (2002) J Am Soc Nephrol 13, 2267
AQP2	Del	p.Glu241fs*31	Kuwahara (2001) Am J Hum Genet 69, 738
AQP2	Del	p.Pro242fs*30	Marr (2002) Hum Mol Genet 11, 779
AQP2	Del	p.Val251fs*21	Sasaki (2013) Clin Exp Nephrol 17, 338
AQP2	Del	p.Gln255fs*15	Kuwahara (2001) Am J Hum Genet 69, 738
AQP2	Del	p.Leu259fs*13	Sasaki (2013) Clin Exp Nephrol 17, 338
AQP2	Del	p.Ala271fs*1	Kuwahara (2001) Am J Hum Genet 69, 738

GENE	Mutation	Amino acid change	Reference
AVPR2	Missense change	p.Gly12Glu	Nossent (2010) J Thromb Haemost 8, 1547
AVPR2	Missense change	P.Ala37Pro	Fujimoto (2014) Yonago Acta Med 57, 85
AVPR2	Missense change	p.Leu43Pro	Vargas-Poussou (1997) J Am Soc Nephrol 8, 1855
AVPR2	Missense change	p.Leu44Pro	Oksche (1994) Biochem Biophys Res Commun 205, 552
AVPR2	Missense change	p.Leu44Phe	Knoers (1994) Kidney Int 46, 170
AVPR2	Missense change	p.Ile46Lys	Pasel (2000) J Clin Endocrinol Metab 85, 1703
AVPR2	Missense change	p.Leu53Arg	Wildin (1994) Am J Hum Genet 55, 266
AVPR2	Missense change	p.Ser54Arg	García Castaño (2015) Eur J Pediatr 174, 1373
AVPR2	Missense change	p.Ser54Arg	Boson (2006) Genet Test 10, 157
AVPR2	Missense change	p.Asn55His	Ala (1998) J Am Soc Nephrol 9, 1861
AVPR2	Missense change	p.Asn55Asp	Wildin (1998) Kidney Int 54, 1909
AVPR2	Missense change	p.Leu57Arg	Ranadive (2009) Clin Endocrinol (Oxf) 71, 388
AVPR2	Missense change	p.Leu59Pro	Ala (1998) J Am Soc Nephrol 9, 1861
AVPR2	Missense change	p.Ala61Val	Birnbaumer (1999) Arch Med Res 30, 465
AVPR2	Missense change	p.Leu62Pro	Knoers (1994) Kidney Int 46, 170
AVPR2	Missense change	p.Arg68Trp	Duzenli (2012) Endocrine 42, 664
AVPR2	Missense change	p.Trp71Term	Holtzman (1993) Hum Mol Genet 2, 1201
AVPR2	Missense change	p.His80Arg	Yuasa (1994) J Clin Endocrinol Metab 79, 361
AVPR2	Missense change	p.Leu81Phe	Wildin (1998) Kidney Int 54, 1909
AVPR2	Missense change	p.Leu83Gln	Knoers (2001) Pediatr Nephrol 16, 1146
AVPR2	Missense change	p.Leu83Pro	Wildin (1994) Am J Hum Genet 55, 266
AVPR2	Missense change	p.Ala84Asp	Albertazzi (2000) J Am Soc Nephrol 11, 1033
AVPR2	Missense change	p.Asp85Asn	Knoers (1994) Kidney Int 46, 170
AVPR2	Missense change	p.Asp85Glu	Sasaki (2013) Clin Exp Nephrol 17, 338
AVPR2	Missense change	p.Val88Met	Bichet (1994) Am J Hum Genet 55, 278

AVPR2	Missense change	p.Val88Leu	Arthus (2000) J Am Soc Nephrol 11, 1044
AVPR2	Missense change	p.Ala89Pro	Böselt (2012) Nephrol Dial Transplant 27, 1521
AVPR2	Missense change	p.Leu90Pro	Sasaki (2013) Clin Exp Nephrol 17, 338
AVPR2	Missense change	p.Gln92Arg	Wildin (1998) Kidney Int 54, 1909
AVPR2	Missense change	p.Leu94Gln	Arthus (2000) J Am Soc Nephrol 11, 1044
AVPR2	Missense change	p.Pro95Leu	Wildin (1994) Am J Hum Genet 55, 266
AVPR2	Missense change	p.Ala98Pro	Cheong (2007) Nephrology (Carlton) 12, 113
AVPR2	Missense change	p.Trp99Arg	Albertazzi (2000) J Am Soc Nephrol 11, 1033
AVPR2	Missense change	p.Arg104Cys	Inaba (2001) J Clin Endocrinol Metab 86, 381
AVPR2	Missense change	p.Phe105Val	Pasel (2000) J Clin Endocrinol Metab 85, 1703
AVPR2	Missense change	p.Phe105Ser	Hong (2014) J Pediatr Endocrinol Metab 27, 93
AVPR2	Missense change	p.Arg106Cys	Bichet (1994) Am J Hum Genet 55, 278
AVPR2	Missense change	p.Gly107Arg	Böselt (2012) Nephrol Dial Transplant 27, 1521
AVPR2	Missense change	p.Gly107Glu	Vargas-Poussou (1997) J Am Soc Nephrol 8, 1855
AVPR2	Missense change	p.Gly107Trp	Duzenli (2012) Endocrine 42, 664
AVPR2	Missense change	p.Cys112Arg	Bichet (1994) Am J Hum Genet 55, 278
AVPR2	Missense change	p.Cys112Tyr	Albertazzi (2000) J Am Soc Nephrol 11, 1033
AVPR2	Missense change	p.Arg113Trp	Bichet (1993) J Clin Invest 92, 1262
AVPR2	Missense change	p.Lys116Asn	Sasaki (2013) Clin Exp Nephrol 17, 338
AVPR2	Missense change	p.Tyr117Term	García Castaño (2015) Eur J Pediatr 174, 1373
AVPR2	Missense change	p.Gln119His	Mori (2017) Clin Exp Nephrol 21, 63
AVPR2	Missense change	p.Gln119Term	Pan (1992) Nat Genet 2, 103
AVPR2	Missense change	p.Gly122Arg	Wildin (1998) Kidney Int 54, 1909
AVPR2	Missense change	p.Gly122Asp	Carroll (2006) Genet Med 8, 443
AVPR2	Missense change	p.Met123Lys	Arthus (2000) J Am Soc Nephrol 11, 1044
AVPR2	Missense change	p.Met123Arg	Sasaki (2013) Clin Exp Nephrol 17, 338
AVPR2	Missense change	p.Tyr124Term	Bichet (1994) Am J Hum Genet 55, 278
AVPR2	Missense change	p.Ser126Phe	Bichet (1994) Am J Hum Genet 55, 278
AVPR2	Missense change	p.Ser127Phe	Arthus (2000) J Am Soc Nephrol 11, 1044
AVPR2	Missense change	p.Tyr128Asp	Vaisbich (2009) Clinics (Sao Paulo) 64, 409
AVPR2	Missense change	p.Tyr128Ser	Pan (1992) Nat Genet 2, 103
AVPR2	Missense change	p.Ile130Asn	Erdélyi (2015) Kidney Int 88, 1070
AVPR2	Missense change	p.Ile130Leu	Boson (2006) Genet Test 10, 157
AVPR2	Missense change	p.Ile-130Phe	Pasel (2000) J Clin Endocrinol Metab 85, 1703
AVPR2	Missense change	p.Leu131Pro	Sasaki (2013) Clin Exp Nephrol 17, 338
AVPR2	Missense change	p.Ala132Asp	Rosenthal (1992) Nature 359, 233
AVPR2	Missense change	p.Leu135Pro	Arthus (2000) J Am Soc Nephrol 11, 1044
AVPR2	Missense change	p.Arg137His	Bichet (1993) J Clin Invest 92, 1262
AVPR2	Missense change	p.Arg137Leu	Feldman (2005) N Engl J Med 352, 1884
AVPR2	Missense change	p.Arg137Gly	Hinrichs (2016) Physiol Rep 4, e12764
AVPR2	Missense change	p.Arg137Cys	Feldman (2005) N Engl J Med 352, 1884
AVPR2	Missense change	p.Ala140Thr	García Castaño (2015) Eur J Pediatr 174, 1373
AVPR2	Missense change	p.Cys142Phe	Mori (2017) Clin Exp Nephrol 21, 63
AVPR2	Missense change	p.Arg143Pro	Tsukaguchi (1993) Biochem Biophys Res Commun 197, 1000

AVPR2	Missense change	p.Arg143Cys	Mori (2017) Clin Exp Nephrol 21, 63
AVPR2	Missense change	p.Trp156Term	Satoh (2008) Endocr J 55, 277
AVPR2	Missense change	p.Leu161Pro	Yamashita (2016) J Pediatr Endocrinol Metab 29, 591
AVPR2	Missense change	p.Val162Ala	Duzenli (2012) Endocrine 42, 664
AVPR2	Missense change	p.Ala163Pro	Rocha (1999) Hum Mutat 14, 233
AVPR2	Missense change	p.Trp164Arg	Sasaki (2013) Clin Exp Nephrol 17, 338
AVPR2	Missense change	p.Trp164Ser	Bichet (1994) Am J Hum Genet 55, 278
AVPR2	Missense change	p.Trp164Term	Albertazzi (2000) J Am Soc Nephrol 11, 1033
AVPR2	Missense change	p.Ala165Asp	Schulz (2002) J Clin Endocrinol Metab 87, 5247
AVPR2	Missense change	p.Ser167Thr	Oksche (1994) Biochem Biophys Res Commun 205, 552
AVPR2	Missense change	p.Ser167Ala	Knoers (2001) Pediatr Nephrol 16, 1146
AVPR2	Missense change	p.Ser167Term	Arthus (2000) J Am Soc Nephrol 11, 1044
AVPR2	Missense change	p.Ser167Leu	Wildin (1994) Am J Hum Genet 55, 266
AVPR2	Missense change	p.Leu169Pro	Huang (2011) J Pediatr Endocrinol Metab 24, 807
AVPR2	Missense change	p.Leu170Pro	Nejsum (2011) NDT Plus 4, 158
AVPR2	Missense change	p.Pro173Leu	Takatani (2010) J Pediatr Endocrinol Metab 23, 415
AVPR2	Missense change	p.Pro173Ser	Albertazzi (2000) J Am Soc Nephrol 11, 1033
AVPR2	Missense change	p.Gln174His	El-Kares (2009) Pediatr Nephrol 24, 1313
AVPR2	Missense change	p.Gln174Arg	Böselt (2012) Nephrol Dial Transplant 27, 1521
AVPR2	Missense change	p.Gln174Leu	Arthus (2000) J Am Soc Nephrol 11, 1044
AVPR2	Missense change	p.Gln180Term	Arthus (2000) J Am Soc Nephrol 11, 1044
AVPR2	Missense change	p.Arg181Cys	Pan (1992) Nat Genet 2, 103
AVPR2	Missense change	p.Gly185Cys	van der Ouweland (1992) Nat Genet 2, 99
AVPR2	Missense change	p.Ser187Arg	Boson (2006) Genet Test 10, 157
AVPR2	Missense change	p.Asp191Gly	Arthus (2000) J Am Soc Nephrol 11, 1044
AVPR2	Missense change	p.Cys192Arg	Gu (2002) Zhonghua Yi Xue Za Zhi 82, 1401
AVPR2	Missense change	p.Trp193Term	Vargas-Poussou (1997) J Am Soc Nephrol 8, 1855
AVPR2	Missense change	p.Trp193Term	Arthus (2000) J Am Soc Nephrol 11, 1044
AVPR2	Missense change	p.Trp200Term	Sakallioglu (2009) J Pediatr Endocrinol Metab 22, 187
AVPR2	Missense change	p.Trp200Term	Schulz (2002) J Clin Endocrinol Metab 87, 5247
AVPR2	Missense change	p.Gly201Asp	Sadeghi (1997) Mol Endocrinol 11, 1806
AVPR2	Missense change	p.Arg202Cys	van der Ouweland (1992) Nat Genet 2, 99
AVPR2	Missense change	p.Arg203Cys	Birnbaumer (1999) Arch Med Res 30, 465
AVPR2	Missense change	p.Thr204Asn	Knoers (1994) Kidney Int 46, 170
AVPR2	Missense change	p.Tyr205His	Sangkuhl (2005) Hum Mutat 25, 505
AVPR2	Missense change	p.Tyr205Cys	van der Ouweland (1992) Nat Genet 2, 99
AVPR2	Missense change	p.Tyr205Phe	Ashida (2007) Pediatr Nephrol 22, 670
AVPR2	Missense change	p.Val206Asp	Knoers (1994) Kidney Int 46, 170
AVPR2	Missense change	p.Thr207Asn	Cheong (1997) Nephron 75, 431
AVPR2	Missense change	p.Trp208Term	Böselt (2012) Nephrol Dial Transplant 27, 1521
AVPR2	Missense change	p.Ile209Phe	Ala (1998) J Am Soc Nephrol 9, 1861
AVPR2	Missense change	p.Phe214Ser	Arthus (2000) J Am Soc Nephrol 11, 1044
AVPR2	Missense change	p.Pro217Thr	Arthus (2000) J Am Soc Nephrol 11, 1044
AVPR2	Missense change	p.Pro217Arg	Hong (2014) J Pediatr Endocrinol Metab 27, 93

AVPR2	Missense change	p.Leu219Pro	Cheong (1997) Nephron 75, 431
AVPR2	Missense change	p.Leu219Arg	van Lieburg (1995) Hum Genet 96, 70
AVPR2	Missense change	p.Gln225Arg	Sasaki (2013) Clin Exp Nephrol 17, 338
AVPR2	Missense change	p.Gln225Term	Knoers (1994) Kidney Int 46, 170
AVPR2	Missense change	p.Phe229Val	Carpentier (2012) J Am Soc Nephrol 23, 1635
AVPR2	Missense change	p.Arg230Pro	Boson (2006) Genet Test 10, 157
AVPR2	Missense change	p.Glu231Term	Holtzman (1994) J Am Soc Nephrol 5, 169
AVPR2	Missense change	p.Glu242Term	Wildin (1994) Am J Hum Genet 55, 266
AVPR2	Missense change	p.Met272Lys	Wildin (1998) Kidney Int 54, 1909
AVPR2	Missense change	p.Thr273Met	Duzenli (2012) Endocrine 42, 664
AVPR2	Missense change	p.Leu274Pro	Schulz (2002) J Clin Endocrinol Metab 87, 5247
AVPR2	Missense change	p.Val277Ala	Wenkert (1994) Hum Mol Genet 3, 1429
AVPR2	Missense change	p.Tyr280Cys	Wenkert (1994) Hum Mol Genet 3, 1429
AVPR2	Missense change	p.Leu282Pro	Albertazzi (2000) J Am Soc Nephrol 11, 1033
AVPR2	Missense change	p.Cys283Tyr	García Castaño (2015) Eur J Pediatr 174, 1373
AVPR2	Missense change	p.Trp284Term	Bichet (1994) Am J Hum Genet 55, 278
AVPR2	Missense change	p.Ala285Pro	Bichet (1994) Am J Hum Genet 55, 278
AVPR2	Missense change	p.Pro286Arg	Pan (1992) Nat Genet 2, 103
AVPR2	Missense change	p.Pro286Leu	Bichet (1998) Proc Assoc Am Physicians 110, 387
AVPR2	Missense change	p.Pro286Ser	Arthus (2000) J Am Soc Nephrol 11, 1044
AVPR2	Missense change	p.Phe287Leu	Chen (2002) J Hum Genet 47, 66
AVPR2	Missense change	p.Leu289Pro	Arthus (2000) J Am Soc Nephrol 11, 1044
AVPR2	Missense change	p.Val290Gly	Schulz (2002) J Clin Endocrinol Metab 87, 5247
AVPR2	Missense change	p.Leu292Pro	Wenkert (1994) Hum Mol Genet 3, 1429
AVPR2	Missense change	p.Trp293Gly	García Castaño (2015) Eur J Pediatr 174, 1373
AVPR2	Missense change	p.Trp293Term	Bichet (1994) Am J Hum Genet 55, 278
AVPR2	Missense change	p.Trp293Term	Wildin (1998) Kidney Int 54, 1909
AVPR2	Missense change	p.Ala294Pro	Ala (1998) J Am Soc Nephrol 9, 1861
AVPR2	Missense change	p.Trp296Term	Kotnik (2007) J Pediatr Endocrinol Metab 20, 483
AVPR2	Missense change	p.Leu309Pro	Shoji (1998) Hum Mutat S1, S278
AVPR2	Missense change	p.Leu309Arg	Albertazzi (2000) J Am Soc Nephrol 11, 1033
AVPR2	Missense change	p.Met311Val	Sahakitrungruang (2010) Horm Res Paediatr 73, 349
AVPR2	Missense change	p.Leu312Term	Bichet (1993) J Clin Invest 92, 1262
AVPR2	Missense change	p.Ser315Arg	Wildin (1998) Kidney Int 54, 1909
AVPR2	Missense change	p.Ser315Arg	Arthus (2000) J Am Soc Nephrol 11, 1044
AVPR2	Missense change	p.Leu316Arg	Sasaki (2013) Clin Exp Nephrol 17, 338
AVPR2	Missense change	p.Asn317Lys	Wildin (1998) Kidney Int 54, 1909
AVPR2	Missense change	p.Asn317Lys	Szalai (1997) Hum Mutat 12, 137
AVPR2	Missense change	p.Asn317Ser	Neocleous (2012) Metabolism 61, 922
AVPR2	Missense change	p.Cys319Arg	Wildin (1998) Kidney Int 54, 1909
AVPR2	Missense change	p.Asn321Lys	Arthus (2000) J Am Soc Nephrol 11, 1044
AVPR2	Missense change	p.Asn321Asp	Arthus (2000) J Am Soc Nephrol 11, 1044
AVPR2	Missense change	p.Asn321Tyr	Wildin (1998) Kidney Int 54, 1909
AVPR2	Missense change	p.Pro322His	Tajima (1996) Pediatr Res 39, 522

AVPR2	Missense change	p.Pro322Leu	Cheong (2007) Nephrology (Carlton) 12, 113
AVPR2	Missense change	p.Pro322Ser	Tajima (1996) Pediatr Res 39, 522
AVPR2	Missense change	p.Trp323Arg	Wildin (1994) Am J Hum Genet 55, 266
AVPR2	Missense change	p.Trp323Arg	Albertazzi (2000) J Am Soc Nephrol 11, 1033
AVPR2	Missense change	p.Trp323Term	Arthus (2000) J Am Soc Nephrol 11, 1044
AVPR2	Missense change	p.Trp323Ser	Szalai (1997) Hum Mutat 12, 137
AVPR2	Missense change	p.Ser329Arg	Faerch (2008) Clin Endocrinol (Oxf) 68, 395
AVPR2	Missense change	p.Ser329Gly	Sasaki (2013) Clin Exp Nephrol 17, 338
AVPR2	Missense change	p.Val332Glu	García Castaño (2015) Eur J Pediatr 174, 1373
AVPR2	Missense change	p.Leu336Pro	Fujimoto (2014) Yonago Acta Med 57, 85
AVPR2	Missense change	p.Arg337Term	Bichet (1994) Am J Hum Genet 55, 278
AVPR2	Missense change	p.Gly352Asp	Albertazzi (2000) J Am Soc Nephrol 11, 1033
